# Imperforate Anus—Operation, &c.

**Published:** 1829-04-01

**Authors:** 


XXVIII.

IMPERFORATE ANUS—OPERATION PERFORMED—FATAL TERMINATION.*

There are many varieties of imperforate anus, some of them much more favourable for a surgical operation than others. It is known that, in some cases, the anus externally seems pervious and well-formed, whilst the rectum is obstructed higher up by a membranous partition. This kind of "imperforation" is generally considered as almost certainly curable by the knife, but the case we are now to detail proves that, under certain circumstances, it is one of considerable danger.

M. Piédagnel, D. M. P. was summoned, on the 15th November last, to visit the infant of Mad. de L. thirty-three hours old. The belly was tense and tumid, the scrotum and penis greatly injected, the breathing hurried, the pulse almost imperceptible, the skin hot and of a violet hue, the face deeper still, the legs and thighs bent upon the pelvis. M. Piédagnel immediately pronounced the little patient to labour under imperforate anus, when the nurses informed him that it had passed but little urine, had had no

---

* Journal Hebdomadaire, No. 10.

stool, and returned an enema as fast as it was given; that it had constantly refused to take any fluid, and had vomited several times. The anus itself was well-formed, but on attempting to introduce a female sound, it was abruptly stopped about an inch up the canal, and could not, by any management, be made to pass beyond that point. The little finger being substituted for the sound, encountered the same obstacle, and the Doctor concluded, without any further hesitation, that the lower portion of the gut ended at this spot in a cul-de-sac, whilst the other was situated at a greater or less height, or separated from the former by a membranous partition.

The only plan was to perform an operation for the child's relief, and in the course of an hour M.P. proceeded to do so, assisted by Dr. Baudelocque, and M.M. Campaignac, Littré, and Reynaud. The general symptoms were but little altered, and that was for the worse; but a swelling was felt above the pubes, which appeared to be the bladder greatly distended. On again introducing the finger in anum, the membrane felt before was thought to be rendered tense by the pressure of fluid above, and presented a kind of fluctuation. A speculum was formed by means of a bit of card rolled up, assisted by dilating the anus by opening the blades of the dressing-forceps, when the extremity of the cul-de-sac was brought into view. A little depression existed in the centre, through which a probe was attempted to be passed in vain. Wishing to avoid all risk of injuring the bladder, which seemed, as was stated above, to be much distended, the catheter was introduced, but no urine flowed, save a few drops in the end of the instrument. Considering that if the bladder was really empty the sought-for object was already gained, and if full, it was located above the pubes, and consequently out of the way, no more attempts with the catheter were made, but the speculum re-introduced into the anus, and the part dilated to its utmost extent. A narrow bistoury was then passed up, the cutting edge turned towards the sacrum, and the point thrust through the little depression, in a direction upwards and backwards. Out of the opening thus made issued a considerable quantity of thick viscous fluid, resembling lees of wine, and containing a greenish matter. It was thought to be a mixture of blood and meconium, and on pressing the abdomen, the quantity that passed from the wound was augmented, and unequivocal meconium was observed. The opening not being large enough to allow free egress to the matters pent up was enlarged towards the left side, by means of the probe-pointed bistoury carried along the groove of a director. The liquid matter issued after this in a large jet, injections of warm water were employed, and the wound on examination was found to be large enough to admit the extremity of the little finger.

The belly diminished in size and relief was experienced, but in six hours afterwards the symptoms were as bad as before. Part of a large elastic gum catheter was introduced into the rectum, and secured there by tapes, when a considerable quantity of fluid, like that observed at first, came away. Soon after the operation the infant had been placed in a warm-bath, and a poultice applied to the abdomen, and at night it was ordered a mixture of oil of almonds and common syrup; at this time there was some, but no material amelioration in the symptoms. Next morning the patient was something better, and on being taken out of a warm-bath, discharged per anum a large quantity of green matters, not tinged with blood. The portion of catheter was removed, and a fold of lint introduced in its stead, but removed also in the course of a couple of hours. In the evening the abdomen was increased in size, especially below, which seemed to be owing to the bladder. No urine had been passed during the day, and several unsuccessful attempts were therefore made to get an instrument into the viscus. Our author thought of puncturing the bladder, either by the rectum or above the pubes, but was deterred by the repugnance of the parents and the general state of the child, who was sinking fast. As will quickly be seen, it was fortunate that no such operation was attempted. Death took place at 7 o'clock that evening, and at 1, P. M. next day the dissection followed, in the presence of the gentlemen formerly named.

On removing the sacrum, the rectum was found to consist of two portions, an upper and a lower. The latter was about an inch in length, of natural breadth, and terminating above in a cul-de-sac, in which was discovered the opening made by the operation. This portion of the gut was composed of the sphincters and the mucous membrane, whilst, towards the cul-de-sac, strong and resisting fibres divided into several bundles and formed a kind of ring, closed, before the operation, by the mucous membrane which had been divided. The upper portion of the rectum was itself divided into two in the following manner. Immediately above the partition which had been punctured, a pouch went off from the rectum, three or four inches in length, and two in breadth, extending along the vertebral column, and filled with blood, partly in a liquid, partly in a coagulated state. This pouch was bounded behind and at the sides by the serous and muscular coats of the gut; in front by the mucous coat, thus torn away from the others, and forming, by such dilaceration, the cavity in which the blood was contained. The other division of this upper portion of the rectum was situated anterior to the pouch above-described, was five inches in length by two or three in breadth, and extended from the umbilicus to within a few lines of the lower portion of the gut. It was greatly distended by air, and ended below in a cul-de-sac, the size of the finger, which presented at its extremity an opening, a line and a half in extent, evidently made by the operation. The mucous membrane, as was mentioned, was detached from the other coats behind and in front; although adherent, the cellular membrane, which formed this union, was more or less filled with clots of blood. All the intestines were greatly distended with gas and green matters, but the rectum contained gas only, and formed that considerable tumour, which, during life, had been mistaken for a distended bladder! This latter viscus was perfectly healthy, and situated behind the pubes and anterior walls of the abdomen.

M. Piédagnel, and the Editors of the Journal Hebdomadaire, have each appended some remarks to the case, which, however, we need not transcribe. It is clear that the operation failed in this instance, from the circumstance of the rectum, above the partition, having been divided, by the separation of its mucous membrane from the other coats, into two distinct cavities, only one of which had been opened into by the bistoury. Had the operator been aware of this, which, of course, he could scarcely be, the patient might not improbably have been saved, by successively opening into both the divisions of the rectum, and giving a free exit to the matters collected above. There cannot be a doubt, we conceive, that the pouch described in the dissection existed before the performance of the operation, although the injection administered afterwards would clearly have tended to enlarge it. M. Piédagnel candidly confesses that, had it not been for the objections of the relatives, and depressed condition of the patient at the time, he would certainly have punctured that tumour above the pubes, which he thought was the bladder, which proved, after death, to be the rectum. The circumstance we only mention to shew the uncertainty of surgery, as well as of physic, uncertainties which, in no country but this, are visited with barbarous denunciations of ignorance on the head of the unfortunate practitioner.

